# Total Mesorectal Excision vs. Transanal Endoscopic Microsurgery Followed by Radiotherapy for T2N0M0 Distal Rectal Cancer: A Multicenter Randomized Trial

**DOI:** 10.3389/fsurg.2022.812343

**Published:** 2022-02-01

**Authors:** Junwei Tang, Yue Zhang, Dongsheng Zhang, Chuan Zhang, Kangpeng Jin, Dongjian Ji, Wen Peng, Yifei Feng, Yueming Sun

**Affiliations:** Colorectal Surgery Division, Department of General Surgery, The First Affiliated Hospital of Nanjing Medical University, Nanjing, China

**Keywords:** radiotherapy, total meso-rectal excision, T2N0M0, rectal cancer, transanal endoscopic microsurgery

## Abstract

**Introduction:**

Transanal endoscopic microsurgery (TEM) is an organ-preserving treatment alternative for patients with early rectal cancer. However, TEM alone is associated with greater risk of local recurrence and inferior survival in comparison with total meso-rectal excision (TME). As an important adjuvant therapy, radiotherapy can effectively reduce the local recurrence rate of rectal cancer. This study aimed to investigate whether TEM followed by radiotherapy can be a valid alternative to TME in T2N0M0 distal rectal cancer treatment.

**Methods:**

We plan to recruit 168 participants meeting established inclusion criteria. Following informed consent, participants will randomly receive treatment protocols of TEM followed by radiotherapy (a total dose of 45–50.4 Gy given in 25–28 factions) or TME. Depending on post-operative pathology, the participants will receive either long-term follow-up or further treatment. The primary endpoint of this trial is 3-year local recurrence rate. The secondary end points include 3-year disease-free survival rate, 3-year overall survival rate, 3-year mortality rate, post-operative quality of life, post-operative safety index, intraoperative evaluation index and post-operative short-term evaluation index.

**Discussion:**

This trial is the first prospective randomized trial to investigate the rectum preserving treatment by using transanal local excision followed by radiotherapy.

**Clinical trial registration:**

The trial was prospectively registered at ClinicalTrials.gov NCT04098471 on September 20, 2019.

## Introduction

Colorectal cancer (CRC) was the third most commonly diagnosed cancer and was the second leading cause of cancer-related death in the world (1). In China, CRC was also one of the most common malignant tumors, and the incidence continues to increase (2), which became a fatal health problem.

Rectal cancer accounts for more than 65% of CRC (3). In the early 1980s, Total meso-rectal excision (TME) was raised by Heald, which emphasized a sharp and meticulous dissection of the tumor and mesorectum with all associated lymph nodes through the avascular embryologic plane (4, 5). TME was considered to the most important progress in surgery for rectal cancer in the last two decades. With the application of TME, the local recurrence decreased to 6 to 12%, and 5-year survival rate improved by 53–87% (6–9). Hence, TME has gradually become a standard component of radical surgery in rectal cancer treatment (10). However, some complications, such as anastomotic leakage, anastomotic hemorrhage, anterior excision syndrome and sexual dysfunction, are common after TME, especially in distal rectal cancer treatment (11–13). Transanal local excision (TLE) is commonly used in benign neoplasms and low-risk superficial malignant rectal cancer. Transanal endoscopic microsurgery (TEM) created by Buess in 1980s, is a technique of TLE which enables the surgeon to perform a full thickness excision with great precision (14). Compared with TME, traditional TLE and TEM both have the significant advantages of preserving anorectal, sexual and urinary functions, reducing the mortality and improving the quality of life (15–17). However, lymph node dissection of rectal cancer was not involved in TLE, which leads to great concern about the increase of tumor recurrence rate and the decrease of survival rate. Although the risk of lymph node metastasis is closely related to the depth of invasion, lymph node metastasis has also been found in patients with early cancer. As reported in the literature, the incidence of lymph node metastasis could reach for 10.3% in T1 stage, 26.1% in T2 stage and 51.2% in T3 stage (18).

Adjuvant therapy for rectal cancer has made great process over the last 40 years, including the adjuvant radiotherapy (RT). Even after the development of the TME with its greatly improved local control rates, radiotherapy significantly decreased the risk of local recurrence (19). For example, the result of a randomized trial of 1,861 patients showed that the rate of local recurrence at 2 years was significantly higher in TME-only group (8.2%) than in TME+RT group (2.4%) (20). The presence of undiagnosed nodal disease, extramural vascular invasion and implantation of cancer cells are the common causes of high local recurrence rate in TLE, whereas radiotherapy can alleviate it by sterilizing the excision bed and adjacent meso-rectal lymph nodes (21). Recently, increasing evidence showed that the organ preservation strategies incorporated RT as an alternative to radical surgery for the early-stage rectal cancer (22, 23). Therefore, for better implementing the organ-preserving strategy, we hypothesized that TEM combined with radiotherapy could be safely and effectively used for the eligible T2N0M0 rectal cancer treatment. The primary objective of this study is to compare the local recurrence rate of TEM followed by radiotherapy and laparoscopic TME surgery in the treatment of T2N0M0 distal rectal cancer. Secondary objectives are long-term survival rate, intraoperative and post-operative situation, and quality of life.

## Methods

### Inclusion Criteria

The inclusion criteria were as followed: (1) Subjects were 18–75 years old; (2) Pre-operative pathological diagnosis was adenocarcinoma; (3) The location of tumor was within 4 cm of anal verge; (4) Tumor size ≤ 3 cm; (5) The mass is mobile and non-fixed; (6) Pre-operative MRI and rectal EUS suggest that the stage was T2 only; (7) Pre-operative high-resolution CT and MRI showed no evidence of lymphatic metastasis or distant metastasis; (8) The general condition of the subjects was fair and their ASA score ≤ 3; (9) Subjects signed an informed consent form.

### Exclusion Criteria

The exclusion criteria were as followed: (1) Participants are Suffering from other malignant tumors within 5 years; (2) Pathological type were poorly differentiated adenocarcinoma, mucinous adenocarcinoma or signet-ring cell carcinoma; (3) Participants are diagnosed as multiple primary colorectal tumors; (4) Participants are pregnant or lactating women; (5) Participants are suffering from severe mental disorders; (6) Participants have Received radiotherapy or chemotherapy already; (7) Participants are suffering from other intestinal diseases (FAP, HNPCC, ulcerative colitis or Crohn's disease); (8) Participants can't suffer abdominal surgery for various reasons; (9) Participants are involved in other clinical trials.

### Exit Criteria

The exit criteria were as followed: (1) Subjects fail for the implementation of the treatment plan for various reasons; (2) The study cannot be continued due to the poor compliance of subjects; (3) Subjects request to withdraw or terminate the treatment due to personal reasons.

### Design and Procedures

**Figure 1:**
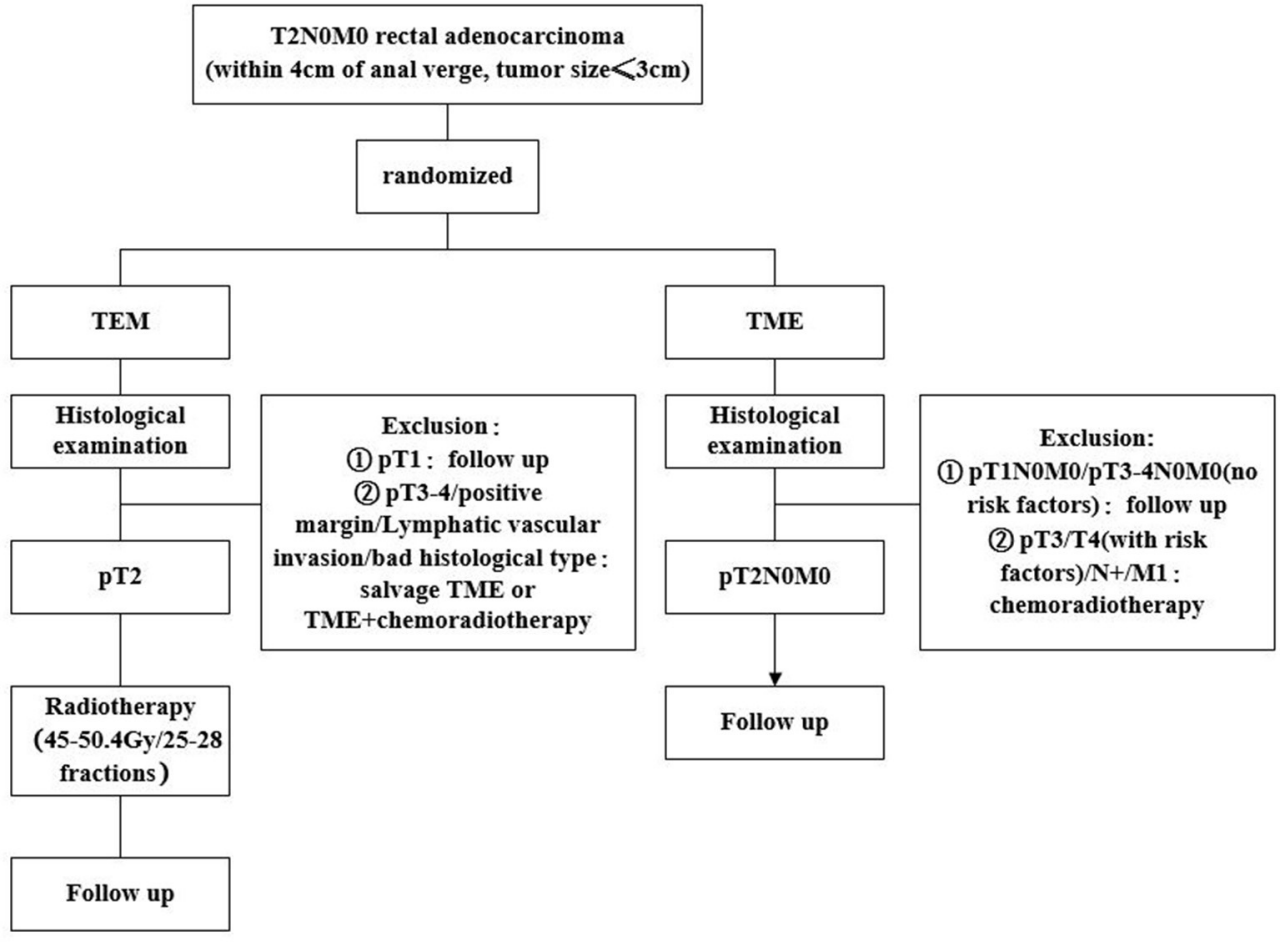
Flowchart of the study.

The study is a prospective, randomized, open and parallel controlled study to determine whether TEM followed by radiotherapy can be considered as an effective therapeutic strategy in eligible T2N0M0 rectal cancer. Firstly, all the subjects who met the inclusion criteria were randomly divided into TME group and TEM followed by radiotherapy group, treated with TME and TEM surgery, respectively. Randomization was performed by an Electronic Data Capture System (EDC) called Clinflash EDC. After histological detection of the resected specimen, subjects undergoing TEM with a pT2N0M0 adenocarcinoma, clear margin and no neurovascular invasion, will receive the post-operative RT with a total dose of 45–50.4 Gy which is given in 25–28 factions. Whereas, subjects with undergoing TME surgery will not receive any other treatment in this situation. Finally, all patients will be followed up. The flow chart of the study is shown in Figure 1.

### End Points

The primary end point is 3-year local recurrence rate. Secondary end points include 3-year disease-free survival rate, 3-year overall survival rate, 3-year mortality rate, post-operative quality of life, post-operative safety index, intraoperative evaluation index and post-operative short-term evaluation index. Specifically, post-operative safety index includes complication rate, perioperative mortality and R0 resection rate. Intraoperative evaluation index includes operative time, intraoperative blood loss, colostomy rate and intraoperative blood transfusion. Post-operative short-term evaluation index includes intestinal exhaust time, post-operative pain and hospital stay. All subjects will be followed up in the outpatient service every 3 months for 3 years. CT scan of abdomen and thorax and colonoscopy must be performed every 6 months. Moreover, ERUS should be performed in patients treated with TEM every 3 months. EORTC QLQ-C30 & LC13 will be measured the quality of life.

### Participating Centers

At least 5 Chinese hospitals will participate in the study, and all of which are experienced in laparoscopic TME and TEM surgery for rectal cancer treatment.

### Sample Size Calculation

In a review of the laparoscopic TME surgery performed in our department, the 3-year recurrence free rate of T2N0M0 rectal cancer patients was nearly 95%. According to 10% of non-inferiority value, 1:1 of the sample size in two groups, 0.025 of the first type of error, 80% power and maximum 10% highest lost rate of follow-up, calculated by Pearson chi-square test, each group needs to include 84 cases, 168 cases in total.

### Statistical Methods

Data collection and analysis will be performed by the SPSS 21.0, Graphpad prsim 5 and Excel software. The measurement data will be expressed as mean ± standard deviation. The statistical analyses will be performed using *t-*tests, Pearson's χ^2^ tests and ANOVA. Cumulative survival analysis will be performed by the Kaplan–Meier method. Differences are considered to be statistically significant at *P* ≤ 0.05.

## Discussion

A TME involves a complete excision of the mesorectum, including associated vascular, lymphatic structures and fatty tissue, is recommended in abdominal resections and significantly improved patients' prognosis (24). With the improvement of oncological outcome, the question has risen if new therapeutic schedule can be developed with safe efficacy and better organ preserving.

Transanal local excision, as a minimally invasive and rectum-preserving treatment, have its advantages of minimal morbidity and mortality, rapid post-operative recovery and a higher post-operative quality of life. Compared with traditional TLE, TEM may be technically feasible for more proximal lesions. A 2015 meta-analysis reported that TEM was oncologically superior to direct TLE for the excision of rectal neoplasms because of a higher rate of negative microscopic margins, a reduced rate of specimen fragmentation and lesion recurrence (25). However, due to the absence of pathologic staging of nodal involvement, the safety of TEM were still controversial for a long time (26–29). At present, TEM is only suggested to treat in selected T1N0M0 rectal cancer or patients physically unfit to undergo TME surgery based on the NCCN guidelines (30).

Although the long-term data on local resection in patients with T2N0M0 rectal cancer are limited, evidence showed that the oncological effect of TLE alone was not satisfactory for patients with T2N0M0 rectal cancer (23, 28, 31, 32). For example, a retrospective study of 1,030 patients showed the 5-year local recurrence rates of T2 tumors increased and the 5-year overall survival decreased after TLE compared to standard resection (31). Even though some reports supported the application of TEM in the treatment of T2 rectal cancer (26, 27), its safety is still worrying (33, 34). For better applying TEM to the treatment of T2N0M0 rectal cancer, TEM combined with adjuvant therapy have been carried out in recent years (35–37). For example, in a prospective multicenter trial named CARTS study (38, 39), patients with a clinical T1-3N0M0 rectal adenocarcinoma were treated with chemoradiation therapy (CRT) for reaching a near pCR, then TEM were performed in patients with good response. The result of 55 patients showed CRT enables organ preservation with additional TEM surgery in approximately two-thirds of patients with good long-term oncological outcome in cT1-3N0M0 rectal cancer. In another prospective multi-institutional trial (40), patients with T2N0M0 rectal cancer were treated with neoadjuvant chemoradiotherapy followed by local excision, the result of 79 patients showed that 3-year disease-free survival and overall survival were 88 and 95%, respectively, suggesting that CRT followed by TLE could be considered as an organ-preserving alternative in selected patients with T2N0M0 rectal cancer. All these findings allow the possibility of saving the rectum by the treatment modality of TEM after neoadjuvant chemoradiotherapy in patients with T2N0M0 rectal cancer. However, many patients cannot accept the strategy of TEM after neoadjuvant chemoradiotherapy and were dying to receive surgical treatment as soon as possible. Additionally, chemoradiotherapy followed by TEM possibly led to a high incidence of complications and gave rise to cumulative toxicities that detract from the benefits of organ preservation (39–42). The strategy of TEM combined with post-operative radiotherapy should be further considered.

Due to the function of adjuvant radiotherapy in sterilizing subclinical mesorectal lymph nodes and the excision bed, the treatment strategy with TLE followed by radiotherapy may be a valid alternative to TME in T2N0M0 rectal cancer (43). In a mutilative analyses of 3,786 patients base on the SEER database (23), survival rates were analyzed between local excision (LE) alone, LE followed by radiotherapy and major resection (MR). The results suggested that the 5-year cancer specific survival rate and 5-year overall survival rate were significantly higher in MR group than those in LE alone group, but similar with those in LE followed by radiotherapy group. However, data is limited and only a few small retrospective studies have reported in this field. To date, there is no prospective randomized study to prove the feasibility of LE followed by radiotherapy in patients with T2N0M0 rectal cancer, further studies are needed in this field.

The trial compared the long-term efficacy of TEM followed by radiotherapy and TME for patients with T2N0M0 rectal cancer, which is the first prospective randomized study in the area. This study will provide strong evidence whether this rectum saving strategy for the treatment of rectal cancer is feasible.

## Data Availability Statement

The original contributions presented in the study are included in the article/supplementary material, further inquiries can be directed to the corresponding authors.

## Ethics Statement

The studies involving human participants were reviewed and approved by the Ethics Committee of the First Affiliated Hospital of Nanjing Medical University. The patients/participants provided their written informed consent to participate in this study.

## Author Contributions

YS, YF, and JT conceived and designed the initial project. The protocol was drafted by YZ, which was modified by DZ, CZ, KJ, DJ, and WP. All authors have read and approved the final study protocol.

## Funding

The authors appreciate the help of all participants that are taking part in the study and the support by Jiangsu Provincial social development key project (BE2021742). Jiangsu Provincial Natural Science Foundation for Basic Research (Grant No. BK20201491), Jiangsu Key Medical Discipline (General Surgery, Grant No. ZDxKA2016005), and the National Key R&D Program of China (Grant No. 2017YFC0908200).

## Conflict of Interest

The authors declare that the research was conducted in the absence of any commercial or financial relationships that could be construed as a potential conflict of interest.

## Publisher's Note

All claims expressed in this article are solely those of the authors and do not necessarily represent those of their affiliated organizations, or those of the publisher, the editors and the reviewers. Any product that may be evaluated in this article, or claim that may be made by its manufacturer, is not guaranteed or endorsed by the publisher.
